# cAMP signalling in trypanosomatids: role in pathogenesis and as a drug target

**DOI:** 10.1016/j.pt.2015.04.014

**Published:** 2015-08

**Authors:** Laura Makin, Eva Gluenz

**Affiliations:** Sir William Dunn School of Pathology, University of Oxford, Oxford, UK

**Keywords:** trypanosomatids, cAMP, phosphodiesterases, drug target, adenylyl cyclases

## Abstract

Despite recent research linking cAMP signalling to virulence in trypanosomatids and detailed studies of trypanosomatid adenylyl cyclases (ACs) and phosphodiesterases (PDEs) since their discoveries 40 years ago, downstream components of the pathway and their biological functions have remained remarkably elusive. However, in recent years, significant discoveries have been made: a role for parasite ACs has been proposed in cytokinesis, evasion of the host immune response, and social motility. cAMP phosphodiesterases PDEB1 and PDEB2 were found to be essential for survival and virulence of *Trypanosoma brucei* and, in *Trypanosoma cruzi*, PDEC2 was shown to be required for normal osmoregulation. As we discuss here, these breakthroughs have led to an ongoing surge in the development of PDE inhibitors as lead compounds for trypanocidal drugs.

## Highlights

• *Trypanosoma brucei* adenylate cyclases are implicated in modulation of host immune response and social motility.
• First effectors downstream of cAMP signalling were identified in *Trypanosoma cruzi* and *T. brucei*.
• Crystal structures reveal a unique pocket in trypanosomatid phosphodiesterases.
• Trypanosomatid phosphodiesterase inhibitors are promising drug candidates.

## Trypanosomatids cause a spectrum of globally important diseases

**Figure 1:**
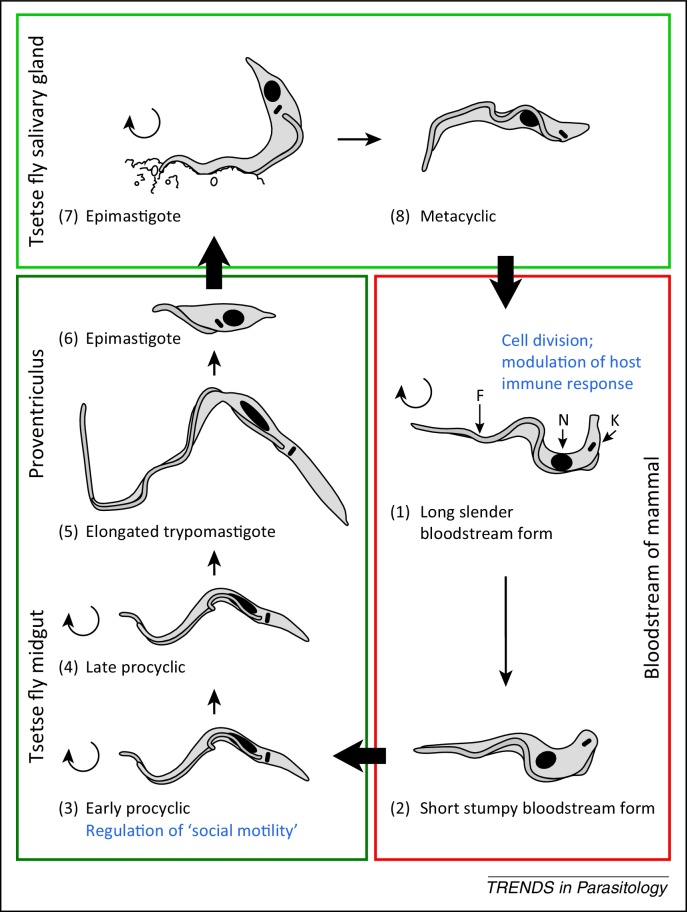
cAMP signalling in the life cycle of *Trypanosoma brucei*. (1) Long slender bloodstream form in the mammalian bloodstream; the position of major organelles is indicated: flagellum (F), nucleus (N), and kinetoplast (K; contains mitochondrial DNA). The adenylyl cyclase (AC) expression site-associated gene 4 (ESAG4) has been implicated in modulation of the host innate immune response [10] and members of the ESAG4 subfamily are important for control of cell division [19]. (2) Short stumpy bloodstream form, which is pre-adapted to differentiate to procyclic forms following ingestion by a tsetse fly. (3) Early procyclic trypomastigote in the tsetse fly midgut. ACs have been implicated in the regulation of social motility [50]. (4) Late procyclic trypomastigote. (5) Elongated trypomastigote, which migrates to the proventriculus. Here, a single asymmetric division produces one long epimastigote, which is thought to decay (not shown) and one short epimastigote (6), which goes on to colonise the tsetse fly salivary glands. (7) Epimastigote attached to the salivary gland. (8) Mammalian-infective metacyclic trypomastigote, which is released from the gland to infect a mammal during a blood meal. Curved arrows denote stages undergoing proliferative cell cycles.

The trypanosomatids are protozoans of the class Kinetoplastida, which includes *Trypanosoma cruzi*, African *Trypanosoma* spp., and *Leishmania* spp.. Subspecies of the African parasite *Trypanosoma brucei* (Figure 1) cause economically important nagana in African cattle as well as human sleeping sickness, which is fatal without treatment in most cases [1]. *Trypanosoma cruzi* is the causative agent of Chagas disease, which can lie dormant for many years before emerging as a severe disease resulting in smooth muscle damage and, in some cases, sudden cardiac death [2]. *Leishmania* spp. cause a spectrum of diseases, ranging from a mild cutaneous form [3] to a lethal visceral form affecting the internal organs [4]. Most of the currently available drugs cannot be administered orally, require long courses of treatment, and do not always result in parasite clearance [1,5,6]. Several of these drugs are associated with unacceptable levels of toxicity: melarsoprol is the only drug available for late-stage *T. b. rhodesiense* infections and is itself the cause of death due to encephalopathy in approximately 5% of patients [7]. Despite the high global health burden caused by trypanosomatids in developing countries and the inadequacy of current treatments, new drugs have been slow to emerge [8].

## Presence of cAMP pathway components in trypanosomatids

cAMP is a second messenger signalling molecule conserved from bacteria to humans and involved in processes as diverse as chemotaxis in *Dictyostelium* and olfaction in mammals [9]. The importance of cAMP signalling in *T. brucei* is hinted at by its high number of genome-encoded ACs, with over 80 genes and pseudogenes (see Glossary) [10] predicted by genome analysis. It remains a mystery why *T. brucei* in particular has evolved to have such a large number of ACs compared with other trypanosomatids. Speculation about putative links between the abundance of AC genes and their functions, such as immune evasion or resolution of multiple incoming signals, could spark interesting research. Observations made over the past 30 years correlating cAMP levels with different life-cycle stages have also pointed towards an important role in development [11]. The discovery that dual knockdown of PDEB1 and PDEB2 in *T. brucei* leads to almost complete inhibition of parasite growth in mouse models [12] was the first definitive link between cAMP signalling and virulence, thereby highlighting cAMP effectors as potential therapeutic targets Here, we summarise recent advances in our knowledge of the role of cAMP signalling in pathogenesis and where we stand with the ongoing development of drugs against parasite PDEs.

## Characterisation of the expression site-associated gene 4 subfamily of ACs in *T. brucei*

**Figure 2:**
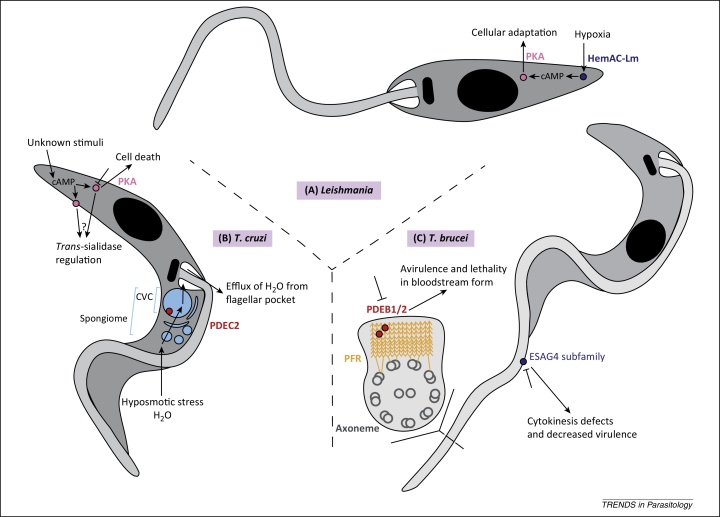
An overview of potential drug targets in cAMP signalling pathways in trypanosomatids. Potential drug targets are highlighted as filled circles and colour coded as follows: pink, protein kinase A (PKA); red, phosphodiesterase (PDE); and dark blue, adenylyl cyclase (AC). **(A)** A cAMP signalling pathway in *Leishmania* mediates cellular adaptation and survival during hypoxia. In *Leishmania major* promastigotes, knockout of heme-containing AC (HemAC-Lm) under hypoxic conditions leads to increased cell death [16]. It is unknown whether HemAC-Lm inhibition leads to cell death in mammalian-stage parasites, but it is possible that HemAC-Lm has a role in adaptation to changes in environmental oxygen in all life-cycle stages by catalysing the production of cAMP, which binds and activates PKA leading to multiple downstream effects. **(B)** cAMP signalling components regulate essential processes in *Trypanosoma cruzi*. Inhibition of PKA in epimastigotes induces cell death [32] and is potentially implicated in immune evasion in the mammalian host by its interaction with *trans*-sialidases in trypomastigotes [33]. PKA localises to both the cytosol and plasma membrane in trypomastigotes. The contractile vacuole complex (CVC) is part of the membranous spongiome network, which is essential for osmoregulation in *T. cruzi*. Inhibition of TcrPDEC2, which localises to the spongiome, in epimastigotes leads to dysregulation of the hyposmotic stress response [54], but it is unknown whether TcrPDEC2 also regulates this process in mammalian-stage parasites. **(C)** PDEB1 and 2 localise to the paraflagellar rod (PFR) and, when simultaneously knocked down, cell death occurs *in vitro* and the parasites are avirulent in mice [12]. ESAG4 subfamily members are flagellar membrane-localised ACs that are highly expressed in bloodstream-form parasites. When the subfamily is simultaneously knocked down, cytokinesis defects occur, leading to cell death and the parasites have attenuated virulence in a mouse model [19].

ACs catalyse the formation of cAMP from ATP. The catalytic domains of class II trypanosomatid cyclases share homology with mammalian class III cyclases, but there are several important distinctions in their structure and proposed mode of action [13]. Whereas the mammalian protein has 12 transmembrane domains, the trypanosomatid ACs have a single transmembrane domain and function as dimers [11,13–15], with the recently described exception of a soluble heme-containing *Leishmania major* AC, HemAC-Lm, which catalyses cAMP production in response to oxygen binding (Figure 2) [16]. Mammalian ACs are indirectly activated by G protein-coupled receptors (GPCRs), which are eukaryotic transmembrane receptors that interact with extracellular ligands to activate heterotrimeric G proteins on the intracellular side of the membrane [17]. Activated GTP-bound G proteins diffuse laterally in the membrane to regulate transmembrane effectors, including AC. Trypanosomatid ACs have an insertion in the G protein-binding domain and the trypanosomatid genomes are thought to contain no heterotrimeric G protein homologues [11]. In contrast to mammalian ACs, the trypanosomatid ACs have large variable extracellular N-terminal domains; this feature suggests direct interaction with extracellular ligands. Despite the intriguing N termini of these ACs, we have come no further in recent years in characterising specific ligands that could activate them.

The *T. brucei* genome contains multiple expression sites (ESs) for a highly expressed variable surface glycoprotein (VSG) that are located downstream of small numbers of co-transcribed genes called expression site-associated genes (ESAGs) [18]. The genome of *T. brucei* encodes approximately 80 AC homologues, some of which are ESAGs [19]. A functionally redundant subfamily of ESAG4 genes includes *ESAG4* itself and two *ESAG4*-like genes, which are non-ES associated [19]. This subfamily is highly expressed in the proliferative slender bloodstream form compared with the cell cycle-arrested tsetse-infective stumpy form of *T. brucei* (Figure 1) [19]. Inducible RNAi knockdown of this subfamily leads to cytokinesis defects with a build up of late cell cycle-stage cells, leading to cell death and decreased virulence in mice (Figure 2) [19]; this was the first phenotype to be characterised upon knockdown of a trypanosomatid AC. Given that VSG is the dominant surface protein in bloodstream-form parasites whose knockdown leads to inhibition of cell cycle progression [20], the authors speculated that the ESAG4 subfamily is involved in sensing aberrant VSG coats as part of a cell cycle checkpoint [19]. This exciting theory remains to be tested.

A further study by the same laboratory implicated ESAG4 in modulation of the host innate immune response [10]. ESAG4 catalytic domain inactivation reduced total parasite AC activity by 50%, despite the apparent redundancy of the ESAG4 subfamily seen in the previous study [10]. These ESAG4 mutants had only slight cytokinesis defects and showed reduced parasitaemia and longer survival of infected mice. This was linked to a reduction of protein kinase A (PKA) activation in host phagocytic cells compared with infections with wild type *T. brucei* [10]*.* Reduced PKA activation resulted in increased tumour necrosis factor (TNF)-α production and inhibition of parasite growth, suggesting a possible role for the parasite AC in regulation of host cell responses [10]. How parasite cAMP or even the transmembrane AC could be translocated from the phagolysosome to act in the host cytoplasm was not addressed. Therefore, it is unclear whether host PKA is activated by cAMP synthesised by ESAG4 or via an indirect, ESAG4-dependent mechanism.

## cAMP is not the signal for *T. brucei* differentiation

Over 30 years of research have linked cAMP levels to differentiation through the life cycle (reviewed in [21]); for example, in the *T. brucei* slender bloodstream form, intracellular cAMP levels are five times higher than in the stumpy form [21,22]. Changes in cellular cAMP level with life-cycle stage have also been observed in *T. cruzi* [23,24] and *Leishmania* spp. [16]. These observations were backed up by the fact that the membrane-permeable cAMP analogue 8-(4-chlorophenylthio)-cAMP could induce slender to stumpy differentiation in *T. brucei* [25]. It is now known that this signal is not cAMP but the products of cAMP hydrolysis [26]. This finding does not necessarily rule out the involvement of canonical cAMP signalling components, because ACs may be necessary for initial cAMP production before hydrolysis if cAMP is the initial signal *in vivo*. In addition, the cAMP effector PKA regulatory subunit (PKA-R) was linked to slender form growth arrest in an RNAi screen [27]. The identification of PKA-R is surprising because *T. brucei* PKA is thought to be cGMP dependent, although the presence of cGMP in *T. brucei* has not yet been confirmed [28]; it is possible that PKA-R is involved in rebalancing purine levels.

## cAMP signalling proteins are important for host–parasite interaction

PKA is a well-known effector of cAMP in mammalian cells, where it phosphorylates a large number of downstream targets to allosterically modulate their activity and induce a cellular response to elevated cAMP levels [29]. A PKA homologue is present in trypanosomatids, although it does not appear to be cAMP dependent in *T. brucei*, where it may have undergone divergent evolution [11,28]. However, PKA is cAMP dependent in *L. major*, where PKA activity was shown to positively correlate with cAMP levels produced by the oxygen-dependent AC HemAC-Lm [16,30]. Knockout of HemAC-Lm under hypoxic conditions resulted in increased cell death [16], suggesting a role for this AC in the survival of the parasite in response to changes in environmental oxygen concentration during its life cycle. *Trypanosoma cruzi* also contains a cAMP-dependent PKA and its inhibition by a genetically encoded PKA inhibitor induced cell death in epimastigotes (Figure 2) [31,32]. Downstream effectors in *T. cruzi* remained unknown until Bao *et al.* performed a yeast two-hybrid screen for TcrPKA catalytic subunit binding partners (Figure 2) [32,33]. They identified members of the *trans*-sialidase superfamily, which are important for evasion of the complement-mediated immune system in the human host as well as attachment to, and invasion of, host cells. Validation of this association by other means would be valuable to exclude the possibility of false positives from the yeast two-hybrid screen.

The first downstream cAMP effectors in *T. brucei* were found by Gould *et al*. in a RNAi screen for PDE inhibition resistance [34]. The putative genes identified were designated cAMP response proteins 1–4 (*CARP1*–*4*). CARP2 and CARP4 are found in the flagellar proteome [35], highlighting the fact that many components of the cAMP signalling pathway, including ACs, PDEs and PKA, are flagellar localised [12,36–38]. The flagellum is an essential organelle and potential drug target, which may serve as a signalling centre for some cAMP-dependent processes [39–41]. CARP1 has homology with mammalian cyclic nucleotide-gated ion channels (CNGs), which are well known as cAMP effectors in mammals [42,43]. In another protist, *Dictyostelium*, a mitogen-activated protein kinase (MAPK) phosphatase conserved in trypanosomatids is involved in the response to cAMP as a chemoattractant [44]. The connection of cAMP and MAPK signalling with proliferation is well known in mammalian cells [45]. In *T. cruzi*, MAPKs have been found to interact with PKA and PDEs in the flagellum [46]; thus, it seems likely that there is MAPK/cAMP signalling crosstalk. Could this be linked with chemoattraction, as in *Dictyostelium*? This process is poorly understood in trypanosomatids, but is thought to be necessary to mediate host–vector transfer and navigation in the insect vector [47].

Swarming behaviour reminiscent of bacteria on a semi-solid agarose surface is an intriguing and rather enigmatic characteristic of early procyclic *T. brucei* [48]*.* Two independent labs have recently proposed this so-called ‘social motility’ to be important in the colonisation of the tsetse midgut (Figure 1) [49,50] and, therefore, it could have an important role in navigation through the fly. ACs were identified as possible candidates for regulation of social motility based on reciprocal upregulation of expression of *AC330* (Tb927.5.285b) and *AC320* (Tb927.5.320) in early and late procyclics, respectively [49]. Subsequently, RNAi knockdown of *AC6* (Tb927.7.7470) or dual knockdown of *AC1* (Tb927.11.17040) and *AC2* (Tb927.10.16190) was found to produce a hypersocial phenotype [50], defined by an increase in the number of swarming groups, implicating these ACs as negative regulators of social motility. This work adds *T. brucei* to the list of microorganisms that use cyclic nucleotide signalling to collectively navigate their environment [51,52]. Interestingly, *AC6* has a flagellar tip localisation and, when knocked down, there is no significant reduction in the gross level of cAMP in the population despite the hypersocial phenotype. Thus, in this case, it is modulation of the local concentration of cAMP rather than the total level in the whole cell that regulates social motility; this hints towards the emerging role of cAMP signalling compartments in the regulation of the cellular response [50].

## PDEs are potential drugs targets

PDEs catalyse the hydrolysis of cAMP to AMP, leading to negative regulation of cAMP signalling. There are four groups of trypanosomatid cAMP PDE (PDEA–D), which are structurally similar to human PDEs [11]. Each group is represented by one or two protein members across the trypanosomatids. Despite their promise as drug targets, the exact functions of trypanosomatid PDEs remain under-researched and most of our knowledge is based on their subcellular localisation [39]. PDEB1 and PDEB2 knockdown in *T. brucei* was found to produce multiflagellated, multinuclear cells that eventually undergo lysis after 42 h and that are essentially avirulent in mouse models (Figure 2) [12]. A clue to the underlying mechanisms of this phenotype may lie in the fact that PDEB1 and PDEB2 have overlapping subcellular localisations: both localise to the paraflagellar rod (PFR), a kinetoplastid-specific structure that lies alongside the flagellar axoneme (Figure 2) and that is essential for normal motility and viability in the mammalian host [35,53]; PDEB2 also has an additional cytoplasmic localisation [12,39].

*Trypanosoma cruzi* PDEC2 (TcrPDEC2) was found to localise to the contractile vacuole complex (CVC) via its phosphatidylinositol (3,4,5)-trisphosphate-binding FYVE domain [54,55], which is essential for both localisation and activity. The CVC is an essential organelle for *T. cruzi* virulence that takes up osmotically active metabolites in response to hyposmotic stress, leading to an influx of water that is later secreted through the flagellar pocket [56]. TcrPDEC2 inhibition led to dysregulation of the osmotic stress response (Figure 2) [54]. The study of *T. brucei* PDEB1 and PDEB2 dual knockdowns in a mouse model provides important proof of principle that dysregulation of cAMP signalling can decrease virulence *in vivo*. In addition, PDEs are required for the normal function of the *T. cruzi* CVC, which is needed for virulence. Taken together, these studies suggest that PDEs are virulence factors and, therefore, promising drug targets.

## Development of drugs against parasite PDEs

PDEBs have been found to be essential proteins in virulence and cell survival [12]; therefore, most drug discovery effort has gone into targeting this group, and TcrPDECs [57] to a lesser extent. The wealth of research in drug development against human PDEs (hPDEs) provides a valuable source of lead compounds and research techniques, and the success of licensed hPDE inhibitors in the clinic [58,59] underlines their promise as drug targets Thus, the field has focussed on altering the specificity of existing hPDE inhibitors and drugs to target trypanosomatid PDEs instead of following the classical drug discovery approach of developing compounds against a unique target in the pathogen. The fact that there are now over 50 published inhibitors for TbrPDEB1 alone suggests that this unconventional approach has been fruitful [60], although it remains to be seen whether good inhibitors will make good drugs. It should be noted that, to produce a lethal phenotype, both PDEB1 and PDEB2 must be inhibited, and that, due to their similar amino acid sequences, it is predicted that an inhibitor designed against one will inhibit both. The high affinity inhibitor CpdA was developed against TbrPDEB1 [61] and induces the same phenotype that TbrPDEB1/2 knockdown produces. Significantly, pre-incubation of *T. brucei* with CpdA led to greatly reduced infectivity upon injection into mice [61].

Crystal structures of TcrPDEC [62], TbrPDEB1 [60], and *L. major* LmjPDEB1 [63] have now been solved and will push forward the discovery of new drugs to fit and inhibit catalytic domains and parasite-specific features. All of these structures confirm the presence of a parasite-specific pocket (P-pocket) next to the active site, which forms a pore through the protein between residues Gln874 and Tyr845. In human PDEs, the pore is gated by two large residues, which are replaced with much smaller residues in the P-pocket. The presence of such a parasite-specific feature is key to the success of drug discovery attempts that target proteins with close human homologues. The TbrPDEB1 P-pocket has been targeted by carrying out fragment growing of existing hPDE-specific compounds into the P-pocket [64] to try and improve the selectivity ratio of these compounds. This technique resulted in compound 20b, which inhibited TbrPDEB1 at nanomolar concentrations. The compound has a selectivity ratio for hPDE of four, which would be unacceptable in a drug [65], but it is already an improvement on previous inhibitors [64]. Indeed, P-pocket binding was found to be an important selectivity driver in an *in silico* screen of a compound library using P-pocket interactions and restrictions imposed by known TbrPDEB1 inhibitors as guidelines [60]. This study found six new TbrPDEB1 inhibitors with the lowest selectivity ratio for hPDE being 0.4. Orrling *et al.* suggest that a selectivity ratio of 10–100 for the parasite enzyme would be desirable in future drug discovery attempts [64]; therefore, these new inhibitors could be good candidates for optimisation. Factors other than selectivity ratio affecting the safety of potential drugs include tolerance of human cells to PDE inhibition and differential effects on specific tissues in the body.

## Concluding remarks

Characterisation of ligands for the extracellular domain of trypanosomatid ACs is likely to be crucial in our understanding of host–parasite interactions and should be a priority (Box 1). For the first time, functions have been assigned to ACs themselves; the possible role of ESAG4 in modulation of the host immune response illustrates the importance of cAMP signalling in host–parasite interactions. Understanding progression through the complex life cycles of trypanosomatid parasites is crucial to enable targeted therapeutic intervention. The first in-depth study of a specific role for cAMP in *T. brucei* differentiation has dispelled the historical view that canonical cAMP signalling is involved. This finding should lead the field to question the involvement of canonical cAMP signalling in other life-cycle switches in trypanosomatids. The study of downstream effectors of cAMP signalling has identified proteins that are crucial for survival in the host, although these represent only a fraction of the total protein component that is likely to be cAMP regulated. The largest body of work in this area has been in drug development. The relatively fast development of many trypanosomatid PDE inhibitors illustrates the value of targeting a parasite protein with homologues that have already been exploited in the treatment of human disorders. Using an existing pool of lead compounds has significantly reduced the research time required for the initial stages of drug discovery, and inhibitors developed over the past couple of years are promising candidates for optimisation. However, we are still far from producing drug candidates due to the inherent problem of achieving high selectivity for the parasite protein using this approach. The drugs with the highest selectivity ratio for the parasite enzyme so far have been produced by targeting parasite-specific features of PDEs, as revealed by recent crystal structure data, and future work should continue this approach.

## Box 1 Outstanding questions

• What are the extracellular signals that activate trypanosomatid ACs? Are there specific ligands for the variable extracellular domains of different ACs?
• Why is there such a large repertoire of ACs in *Trypanosoma brucei*?
• What is the mechanistic link between *T. brucei* AC activity and cytokinesis? Is cytokinesis failure a direct or indirect consequence of the ablation of ESAG4 and are aberrations in the VSG coat sensed through ESAG4?
• What is the mechanistic link between *T. brucei* AC activity and ‘social motility’? Do specific flagellar ACs modulate the beat pattern of the flagellum, impacting on directional motility? Can factors produced by one trypanosome activate ACs in another?
• Is PKA in cells of infected animals activated directly by ESAG4-generated cAMP? How might *T. brucei*-generated cAMP or even the transmembrane AC itself be translocated from the phagolysosome to act in cells of the infected animal to modulate immune responses?
• If the *T. brucei* PKA homologue is not activated by cAMP, does another protein fulfil its function as a master regulator of downstream effectors of cAMP signalling?
• Will targeting the P-pocket of trypanosomatid PDEs produce drug candidates with high enough selectivity for parasite versus human PDEs to be safe and effective drugs?

## Glossary

*Dictyostelium*: a nonpathogenic, soil-dwelling amoeba whose ability to form multicellular ‘slugs’ has made it a model organism for studying the collective behaviour of single-celled organisms and chemotaxis using cAMP.
Expression sites: loci found at the telomeres of large and intermediate chromosomes of *Trypanosoma brucei.* Polycistronic transcription of VSG and ESAGs occurs here and is restricted to one expression site at a time.
Phagolysosome: the compartment in phagocytic cells that is the result of fusion between the phagosome and lysosomes. Protozoan parasites will either be degraded here or, as is the case for *Leishmania* spp., may go on to make this compartment their intracellular niche for replication.
Pseudogenes: genomic DNA sequences related to protein-coding genes but which have lost their ability to encode proteins due to mutations.
Selectivity ratio: the selectivity ratio for a drug is defined as the ‘IC_50_ for off-target enzyme/IC_50_ for target enzyme’, where the IC_50_ is the half-maximal concentration of drug needed to inhibit the enzyme. The higher the ratio, the more selective the drug is for the target enzyme.
*Trans*-sialidases: this large family of *Trypanosoma cruzi* enzymes transfers alpha(2-3)-linked sialic acid from sialoglycoconjugates on the host cell surface to an acceptor on the parasite surface, thereby helping to disguise the parasite with a molecule that the immune system recognises as self.
Tsetse fly: a large fly (genus *Glossina*) that is found across mid-continental Africa. It lives on the blood from vertebrate animals, including humans, and is responsible for transmitting African trypanosomes (*T. brucei*, *Trypanosoma congolense*, and *Trypanosoma vivax*).
